# Curling of Gel Scaffold Layer for Cell Culture by a Deformable Microactuator Mat Toward Biological Canal Formation

**DOI:** 10.3390/mi16091019

**Published:** 2025-09-03

**Authors:** Satoshi Konishi, Shiho Shimizu, Katsunori Sakai

**Affiliations:** 1 Department of Mechanical Engineering, College of Science and Engineering, Ritsumeikan University, Kusatsu 525-8577, Japan; 2 Graduate Course of Science and Engineering, Ritsumeikan University, Kusatsu 525-8577, Japan; 3 Ritsumeikan Advanced Research Academy, Kyoto 604-8520, Japan; 4 Ritsumeikan Global Innovation Research Organization, Ritsumeikan University, Kusatsu 525-8577, Japan

**Keywords:** biological canal formation, deformable microactuator mat, pneumatic balloon actuator, perfusion test, permeation test

## Abstract

A gel scaffold for a biological canal is formed using a deformable soft microactuator mat. Three-dimensional cellular tissue structures are important for organ-on-a-chip in in-vitro biomimetic models. However, most traditional cellular tissues have been cultured in a dish or transwell. Furthermore, cellular culture on the inner wall of pre-manufactured channels has been recently reported. In this study, we propose a deformable actuator mat that can transform a flat structure into a tubular structure. The active mat, which is composed of pneumatic balloon actuator arrays, assembles a biological canal from a flat sheet of a gel scaffold for cell culture. The mat can return to its initial flat state so that the gel-based canal structure with cells can self-stand. A self-standing tubular gel structure is demonstrated as a biomimetic canal toward a biological canal with cells. A self-standing tubular gel structure has permeability, which is important for evaluation of pharmacokinetics. The actuator mat under the gel layers was curled into a tubular shape (approximately 1 mm diameter) and returned after the assembly. Culturing cellular tissues on a demonstrated gel structure will reproduce the biological permeability of organs such as an intestinal tract. This study confirms the gel-based canal formation process without cells as a feasibility study. The proposed technique has potential for the flexible design of biological three-dimensional structures, thereby contributing to pharmacokinetics research.

## 1. Introduction

The remarkable progress in tissue engineering has contributed to the development of regenerative medicine and drug discovery. Micro-total analysis systems integrated with microfluidic devices have been developed for lab-on-chip applications [1]. As a result of lab-on-chip cell culturing, organ-on-a-chip (OoC) has emerged [2]. OoCs prepared from tissues and organs on a chip can be used for drug screening as an alternative to traditional animal testing. Various OoCs, such as heart-on-a-chip [3], lung-on-a-chip [4], and gut-on-a-chip [5], have been developed by culturing cells on a chip. In particular, the OoC for the small intestine, which is categorized as a gut-on-a-chip, is important for pharmacokinetic drug screening. Cells were cultured on a porous membrane, and intestinal permeability was reported [5]. Flow through the mimicked small intestine can apply shear stress to cells and drug transportation [6]. Moreover, the scaffold mimicked a real small intestine [7], and peristaltic motion was generated for accurate evaluation [8].

Cell morphology and intercellular connections are affected by scaffold stiffness [9]. ECM has been used as a scaffold for cell culture [10,11]. The transepithelial electrical resistance (TEER) depends on the cell culture status, including density and thickness. The integration of electrodes into OoCs can improve the utility of TEER measurements for real-time noninvasive monitoring [12].

Recently, tissue engineering has required a three-dimensional cellular structure for improving biomimetic modeling, whereas cultured cells are conventionally planar in dishes or microwells. Here, we report an openable artificial small intestinal tract as a transformable OoC, or gut-on-a-chip [13]. The proposed openable OoC can be transformed from a flat shape into a tubular shape using integrated actuator arrays. Pneumatic balloon actuators (PBAs) made of polydimethylsiloxane (PDMS) were used to close and open the tubular structure. Cells were cultured on actuator arrays, which served as the inner wall of the closed artificial small intestine. The cells were observed and sampled when the artificial small intestine was opened. Drug absorption by Caco-2 cells was evaluated using the developed device [14]. Peristaltic motion was performed using a tubular device. The PBA arrays were sectioned for individual motion control to generate peristalsis in a tubular structure [15].

**Figure 1:**
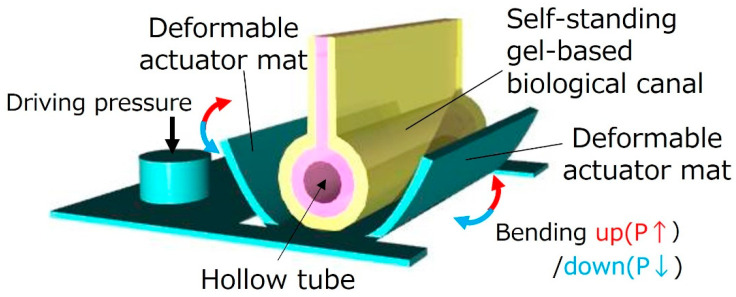
**Biological canal formation by gel scaffold**. The gel is hardened on the artificial intestinal tract device in a flat state. After culturing the cells, the device is driven into a tubular state, and the top of the gel is fixed using a jig. Syringes are connected to silicon tubes inserted at both ends of the gel tube device to circulate the drug solution. A gel tube device consists of two layers of gel. The cell culture layer is collagen gel, and the base layer is agarose gel with easily adjustable stiffness. The device can be opened and closed by pressure control.

Figure 1 illustrates biological canal formation using a deformable actuator mat. As a feasible study toward the biological canal formation, this study demonstrates a gel scaffold canal formation without culturing cells to confirm the feasibility of the proposed process. The proposed method will be applied to biological canal formation by culturing cells. In fact, cells were successfully cultured on a similar structure in our previous works [13,14]. The actuator mat, which is composed of an array of bending PBAs, exhibits the same structure as an openable artificial small intestine tract system. Previous studies on an openable artificial small intestine tract system have used the actuator as a part of its structure. Our previous OoC with actuators was integrated with external filters for permeability to evaluate pharmacokinetics [16]. On the other hand, a self-standing tubular gel structure by detaching an actuator mat has permeability, which is important for evaluation of pharmacokinetics. The method proposed in Figure 1 uses an actuator mat as an assembly tool to form tubular structures of gel films with cultured cells. The actuator mat, which is normally flat on a substrate, bends up by pressurizing PBAs in the actuator mat. It bends down by decompressing PBAs and finally returns to an initial flat state. The self-standing gel-based biological canal is released from the actuator mat after the formation process is completed.

## 2. Materials and Methods

### 2.1. Deformable Actuator Mat for Assembling Gel-Based Biological Canal

**Figure 2:**
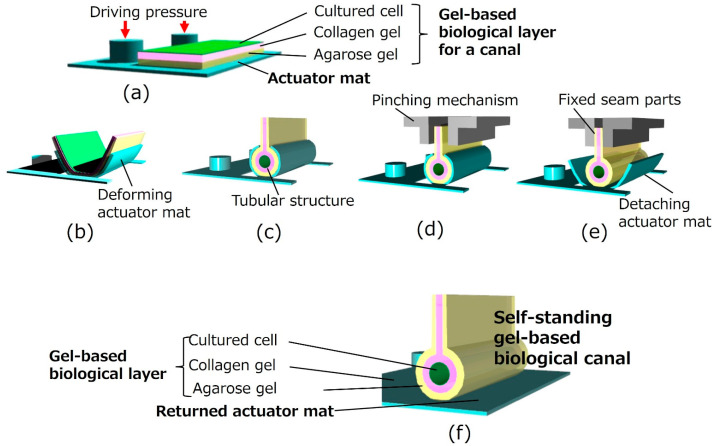
**Formation of a gel-based biological canal by a deformable actuator mat**. (**a**) **Initial flat state**. Thee agarose gel solution and the collagen gel solution are prepared on the actuator mat. Cells are seeded on the gel. (**b**,**c**) **Transformation from flat to tubular structure**. The deformable actuator mat is transformed into the tubular state. (**d**) **Pinching and fixing seam parts**. Both ends of the gel layers, as well as seam parts of the gel-scaffold film, are fixed together by a pinching mechanism of the external jig. (**e**) **Detaching actuator mat**. A gel-based biological canal is detached for self-standing by decompressing the actuator mat. (**f**) **Completed gel-based biological canal**. A silicon tube is inserted and connected to both ends of the gel-based biological canal. The drug solution can be circulated for evaluation through the completed gel-based biological canal.

**Figure 3:**
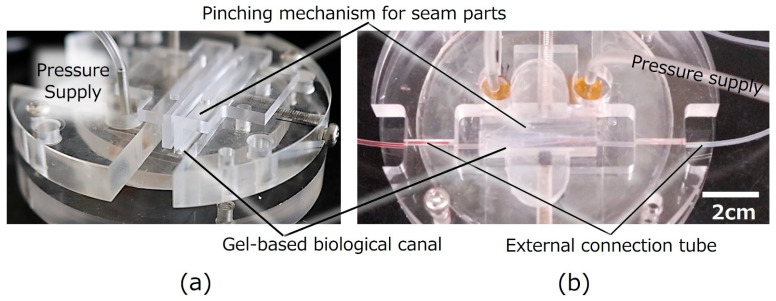
**Setup image of the external jig with a pinching mechanism**. (**a**) **Pinching mechanism for seam parts of gel-scaffold film**. Both ends of the gel layers as seam parts of the gel-scaffold film, are fixed together by a pinching mechanism of the external jig. (**b**) **Setup for perfusion test**. An external silicone tube was inserted and connected to both ends of the gel-based biological canal as the inlet and outlet interconnections.

Figure 2 illustrates the assembly method for the proposed biological canal formation. The gel-based biological layers, which are composed of cultured cells on gel layers, were formed on an actuator mat composed of pneumatic balloon actuator (PBA) arrays as depicted in Figure 2a. The deformable actuator mat was transformed into a tubular state, as shown in Figure 2b,c. The PBA arrays integrated into the actuator mat were pressurized and bent using a syringe pump (PUMP33; Harvard Apparatus, Holliston, MA, USA) to form a tubular structure. After packaging an external jig for fixation and evaluation equipment, the gel-based biological canal was detached for self-standing by decompressing the actuator mat (Figure 2d,e). Both ends of gel layers as seam parts are fixed together by a pinching mechanism of the external jig during the process in Figure 2d,e. The external jig will be explained later in Figure 3. Figure 2e illustrates a completed gel-based biological canal assembled by the actuator mat of PBAs.

The deformable actuator mat of PBAs, mounted on an acrylic system platform structure, was mainly composed of PDMS (Silpot 184, Dow Silicones Corp., Midland, MI, USA). Prepolymer ratios of 8:1 and 12:1 were used for the inflatable and base layers, respectively. The PDMS microstructures were cast using a mold patterned through the photolithography of a thick negative photoresist (product number: SU-8 3050; Newton, MA, USA). The molded PDMS structures were bonded and integrated to complete the fabrication process. A further detailed fabrication process for the actuator mat was reported in a previous publication on the artificial intestinal tract system [13].

### 2.2. Gel-Based Scaffold Formation

Gels for both culture scaffolding and structural materials were selected based on their biocompatibility for cell culture and stiffness. Gel layers were composed of agarose gel and collagen gel in this study. The gel-based biological layer, together with a deformable actuator mat, should be sufficiently flexible to bend and transform into a tubular shape. Simultaneously, the gel-based biological canal stands when detached by decompressing the actuator mat. Agarose gel (Nacalai Tesque, Kyoto, Japan) was selected as the outer structural layer owing to its high stiffness for self-standing. A collagen gel based on Cellmatrix Type I-A (Nitta Gelatin Inc., Yao, Japan) was employed as the inner scaffolding layer because of its high biocompatibility. Gel specimens 9 mm in diameter and 8 mm in height were prepared, and their stiffness was estimated. The specimens were pressed with 400 μm (strain 0.05), and the stress was measured by a load cell (LVS-2KA, Kyowa Electronic Instruments Co., Ltd., Tokyo, Japan) for estimating Young’s modulus. Agarose gels with 1 wt% and 5 wt% and a collagen gel exhibited values of 24.5 kPa, 139 kPa, and 541 Pa, respectively. As a result of the stiffness estimation, a 200 μm-thick collagen gel was coated on a 200 μm-thick agarose gel (1 wt%). The total bending rigidity of the gel-based biological canal with the actuator mat was designed to be below 7.0 × 10^−11^ Pa × m^4^, as decided through a preliminary experiment on deformability. The agarose gel solution was dropped onto an actuator mat, and the acrylic mold was placed using a 3D printer (AGILISTA, Keyence, Osaka, Japan) and left at room temperature for 5 min. After curing the agarose gel layer, the collagen gel solution was poured and incubated at 37 °C for 1 h. After curing all gel layers, the cells were seeded on the collagen gel. This paper deals with a deformable soft microactuator mat for a gel scaffold without cells toward a biological canal. Cell culture on a proposed device will be planned for future work. In preparation for the cell culture on the device toward the biological canal, we have confirmed the following sterilization process. After UV irradiation sterilization for 0.5 h in the bio clean bench (MCV-B91F, SANYO, Tokyo, Japan), the device is cleaned by using PBS (D-PBS(-) without Ca and Mg, liquid, Nacalai Tesque, Kyoto, Japan).

### 2.3. Gel-Based Biological Canal Formation for Evaluation of Perfusion and Permeation

The gel-based biological layers, composed of cultured cells on gel layers, were deformed by the actuator mat and released for self-standing as explained in Figure 2. The assembled self-standing canal was observed under a digital microscope (VHX-500F; Keyence, Osaka, Japan). Figure 3 shows the experimental setup of a gel-based biological canal together with an external jig, which is for fixation and evaluation. The assembled canal was implemented by the external jig for the pinching mechanism and implementing evaluation instruments. Seam parts of gel layers are fixed by a pinching mechanism of the external jig in the process of Figure 2d,e. After assembling, an external silicone tube was inserted and connected to both ends of the gel-based biological canal as the inlet and outlet interconnections (Figure 3a). The drug solution was pumped (PUMP33; Harvard Apparatus, Holliston, MA, USA) and circulated through the tubes connected to the gel-based biological canal for perfusion evaluation. The drug permeation test used uranine dye (NACALAI TESQUE, INC., Kyoto, Japan), which was adjusted (25 μM) and introduced into the gel tube at 0.05 mL/min. Samples were estimated by a fluorescence microplate reader (Synergy HTX, Agilent Technologies Japan, Ltd., Tokyo, Japan).

## 3. Results and Discussion

### 3.1. Deformable Actuator Mat Performance for Curing Gel Scaffold Film

**Figure 4:**
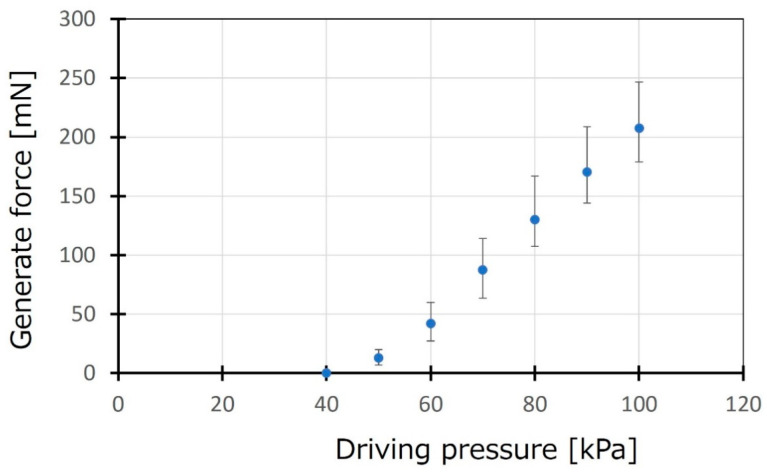
**Evaluation of generated force by the actuator mat**. The generated force by the actuator mat was evaluated for an array of 60 arrays of PBA. A load cell sensor was set at 1 mm high from the initial position of the tip of the actuator mat.

A deformable actuator mat consists of PBA arrays and transforms into a tubular state by pressurizing for curling gel scaffold on the actuator mat. Generated force by the actuator mat was evaluated to guarantee its ability of curling gel scaffold composed of agarose gels coated by a collagen gel. Figure 4 shows measurement results of generated force by an array of 60 PBAs. The tip of the actuator mat deformed and contacted a load cell sensor, which was set at 1 mm high. The load cell sensor began to detect the generated force when applied pressure increased over 40 kPa. The average force generated by the actuator mat was estimated to be approximately 200 mN at 100 kPa, whereas a single PBA generated more than 3 mN. The result could guarantee gel-based canal formation by the actuator mat.

### 3.2. Self-Standing Gel-Based Canal Formation by a Deformable Actuator Mat

**Figure 5:**
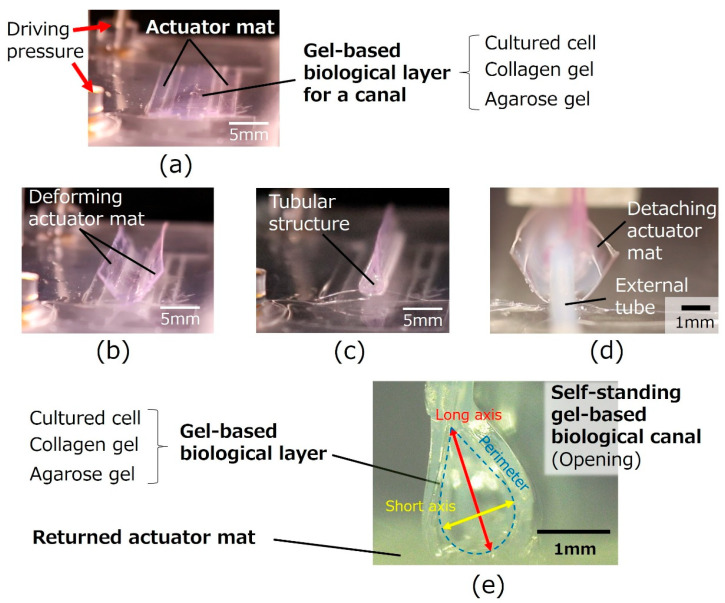
**Self-standing gel-based canal formation by a deformable actuator mat**. (**a**) **Initial flat state**. Drop the agarose gel solution onto the artificial intestinal tract device, put the mold on it, and leave it at room temperature for 5 min. Typical thickness is 200 µm. Next, drop the collagen gel solution and incubate at 37 °C for 1 h. Cells are seeded on the gel. (**b**,**c**) **Transformation from flat to tubular structure**. The deformable actuator mat is transformed into the tubular state. (**d**) **Detaching actuator mat**. A silicon tube is inserted and connected to both ends of the gel-based biological canal. The drug solution can be circulated for evaluation through the completed gel-based biological canal. A gel-based biological canal is detached for self-standing by decompressing the actuator mat. (**e**) **Completed gel-based biological canal**. The end-face view of the self-standing gel-based canal was depicted. The short axis and perimeter are approximately 1 mm and 3.5 mm after detaching.

A gel-based canal was formed via the proposed method using a deformable actuator mat. Figure 5a–e, which corresponds to Figure 2a–e, shows the sequential process of the formation of a self-standing gel-based canal. A completed self-standing gel-based canal was shown in Figure 5e. The deformable actuator mat was transformed into a tubular shape (1 mm diameter) at an applied pressure of 70 kPa. The pressurization for driving the actuator mat was stopped after the top ends of the gel layers were fixed by an external sealing mechanism. The actuator mat returned to its initial flat shape, and the gel-based canal was detached. The gel-based canal was positioned along with the interconnection tubes at its inlet and outlet. Figure 5e shows an end-face view of the gel-based canal. The opening section of the canal was not a true circular shape but rather a flat shape, where the interconnection tubes were removed on account of the observation. The short and long axes and perimeter of the opening section of the canal were defined and evaluated. The short axis and perimeter were approximately 1 mm and 3.5 mm after detachment.

### 3.3. Perfusion and Permeation Test Through Gel-Based Canal

**Figure 6:**
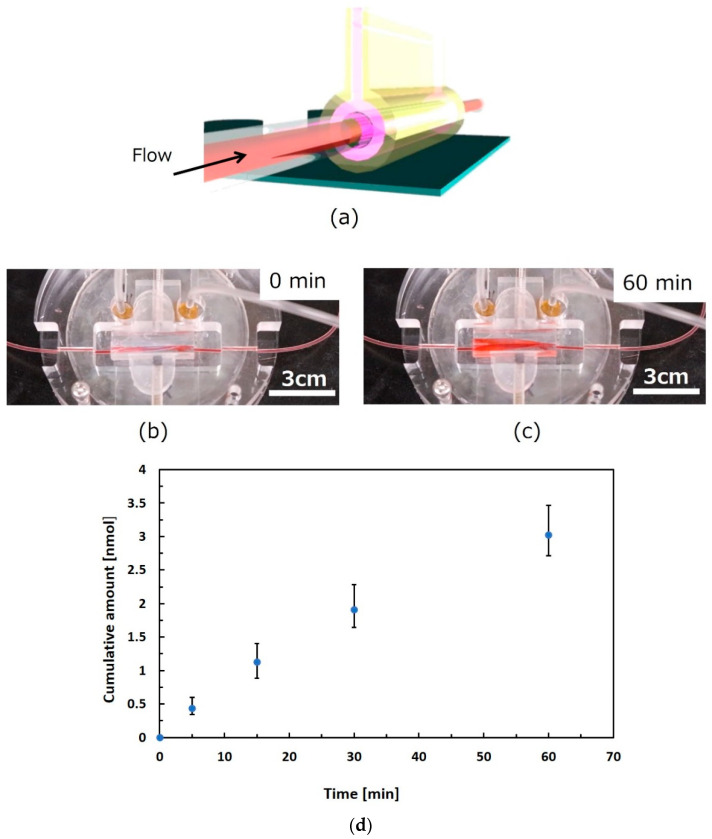
**Perfusion test through gel-based canal**. (**a**) **Schematic view of setup for perfusion test**. (**b**) **Gel-based canal packaged with peripheral structures**. (**c**) **Observation of flowing water in the canal using a red dye compound**. (**d**) **Evaluation of substance permeation through the gel**. We left the gel tube in water for 60 min and confirmed that the change in the perimeter was not obvious. Observation of the behavior of flowing water in the canal was executed at a flow rate of 0.05 mL/min for 60 min. The colored water was pumped for 60 min (0.05 mL/min), and no unexpected leakage was confirmed. The gel absorbed the colored water and was dyed red after 60 min. The uranin dye for visualization was used for permeated substances for evaluation of substance transmission. The uranine was introduced into the gel tube at a flow rate of 0.05 mL/min. Fluorescence intensity of permeated samples was evaluated.

A perfusion test of the liquid, assuming that culture media and drugs passed through the gel-based canal, was performed by using the setup illustrated in Figure 6a. Figure 6b shows a complete view of the gel-based canal packaged with peripheral structures, including interconnection tubes and sealing mechanisms. In advance of the perfusion test, the shape-retention property of the gel-based canal was evaluated in water for 60 min in terms of the liquid absorption and permeation. Perfusion time was also determined in terms of the general lag time of permeation from the inner to the outer part of the biological canal. Both the axes and perimeter of the opening section of the canal were maintained through a water absorption test for 60 min. The expansion and contraction of the cell culture surface due to water absorption by the gel were not large. The gel-based canal did not show an obvious change in shape during the water absorption and permeation tests. New coccine (FUJIFILM Wako Pure Chemical Corporation, Osaka, Japan) was used as a red dye compound for coloring of water for observing behavior of flowing water in the canal as shown in Figure 6b,c. The water flow behavior was observed at a flow rate of 0.05 mL/min for 60 min by pumping (PUMP33, Harvard Apparatus, Holliston, MA, USA). No unexpected leakage was confirmed, whereas the gel was dyed red owing to the absorption of colored water 60 min after the perfusion test (Figure 6c). Following the preliminary results, liquid permeation was evaluated. Figure 6d evaluated permeated substances through the gel in accordance with time, where the uranine dye was used as a permeated substance for visualization. The uranine was introduced into the gel tube at a flow rate of 0.05 mL/min. Fluorescence intensity of permeated samples was evaluated. As a result, the accumulated amount of permeated uranine increased in accordance with the time.

## 4. Conclusions

This study proposed a deformable actuator mat for transforming a flat structure into a three-dimensional structure. The proposed technology enabled a biological canal by transforming a flat gel scaffold with cultured cells. The active mat made of polydimethylsiloxane assembled a biological canal by driving its pneumatic balloon actuators. The mat returned to its initial flat state so that a gel-based canal structure with cells could be formed. The typical diameter of the canal was approximately 1 mm. In reality, the current cross-sectional shape of the canal is not a perfect circle. Improving the shape as well as evaluating the influence of imperfect circles in terms of shear stress by flow in the canal are ongoing. Assuming that culture media and drugs passed through the gel-based canal, a perfusion test was performed by using a gel-based canal structure. The water flow could be operated through the gel-based canal at a flow rate of 0.05 mL/min for 60 min. Substance permeation through the gel was also evaluated in the perfusion test. Fluorescence intensity of permeated uranine increased in accordance with the time. This study confirmed the feasibility of the biological canal formation process by using a gel scaffold without culturing cells. As prospects, biological canal formation with cultured cells such as MDCK cells and Caco-2 cells is our next interest. Consequently, the proposed gel-based biological canal has potential for the flexible design of biological three-dimensional structures, especially for pharmacokinetics research using cultured cells.

## Data Availability

All data generated or analyzed during this study are included in this published article.
